# Humoral immune response after SARS-CoV-2 vaccination in cladribine-treated multiple sclerosis patients

**DOI:** 10.1016/j.jvacx.2024.100445

**Published:** 2024-01-20

**Authors:** M. Mimpen, D. Kreiter, T. Kempkens, S. Knippenberg, R. Hupperts, O. Gerlach

**Affiliations:** a Academic MS Center Zuyderland, Zuyderland Medical Center Sittard-Geleen, the Netherlands; b School for Mental Health and Neuroscience, Department of Neurology, Maastricht University Medical Center, Maastricht, the Netherlands

**Keywords:** Multiple sclerosis, COVID-19, Cladribine, Vaccination

## Abstract

Multiple sclerosis immunomodulatory treatments such as cladribine, which affects both B- and T-lymphocytes, can potentially alter the humoral response to SARS-CoV-2 vaccination. This monocenter retrospective study reports on anti-SARS-CoV-2 IgG antibody response in cladribine treated MS patients and we compare the response in patients vaccinated before and after an 18-week interval after last cladribine dose. Of the 34 patients (5 patients ≤ 18 weeks and 29 patients > 18 weeks after last cladribine dose) that were included, 32 reached seropositivity (94 %). All patients vaccinated < 18 weeks after last cladribine dose reached seropositivity. This study confirms findings of earlier reports that cladribine-treated MS patients show an adequate humoral response after SARS-CoV-2 vaccination, even when vaccinated early (≤18 weeks) after last cladribine dose.

## Introduction

Severe acute respiratory syndrome coronavirus-2 (SARS-CoV-2) is a highly contagious airborne virus which causes coronavirus disease 2019 (COVID-19) [1]. As most multiple sclerosis (MS) patients suffer from some form of immune alteration due to disease-modifying treatment (DMT), the effects of COVID-19 infection in MS patients have been a topic of great interest during these last two years [2], [3].

Similarly, as DMTs alter the immune system of a MS patient, the effects of a COVID-19 vaccination in this population are of interest as well. Patients with immunosuppressive therapies were excluded from the larger trials and, as such, clinicians and patients are reliant on case reports and smaller studies to evaluate the effects and success rate of COVID-19 vaccination. Early publications appear positive in most therapies, although a reduced immunization has been reported in patients using anti-CD20 therapies (ocrelizumab, rituximab), as well as fingolimod [4], [5], [6], [7], [8].

Cladribine is a relatively new DMT used in the treatment of relapsing-remitting (RR)MS. Cladribine is a purine analogue, which accumulates primarily in lymphocytes and halts DNA synthesis, leading to cell death in both dividing and resting lymphocytes [9]. It affects B cells more than T cells, both of which are instrumental in a successful immunization [10]. Because of the immunosuppressive effect and scarcity of data, international and national workgroups advised clinicians to be restrictive in prescribing cladribine during the pandemic. Consequently, data on the humoral response to SARS-CoV-2 infection in cladribine treated MS patients is limited. Several studies report successful humoral responses to both infection and vaccination, with nearly all cladribine treated patients reaching seropositivity after vaccination (cumulative sample size of 208 patients) [4], [11], [12], [13], [14], [15], [16], [17]. Regarding vaccination, most workgroups advised vaccination at least 4 weeks before next dose and a minimum interval varying between 12 and 18 weeks after the last dose, depending on the country [18], [19]. Later, among others, the MS International Federation abandoned the minimum interval between vaccination and last cladribine dose, as the evidence to date did “not suggest that timing the vaccine in relation to cladribine dosing is likely to make a significant difference” [18].

In this real-world study, we investigate the humoral response to several COVID-19 vaccinations in MS patients using cladribine, measured as SARS-CoV-2 antibody levels in serum, with the aim to extend on and assess reproducibility of the results from earlier studies. As a secondary objective, we aim to investigate the relationship between time since last cladribine cure and vaccination response.

## Methods

In February 2022, we retrospectively included patients from the outpatient clinic of Academic MS centre Zuyderland (Sittard-Geleen, The Netherlands) who were treated with one or more courses of cladribine tablets and in which the antibody response against SARS-CoV-2 was determined after vaccination or infection as part of routine clinical care. The last cladribine tablet course had to be less than 2 years before the first vaccination. Patients that received at least one vaccination (with or without having endured a SARS-CoV-2 infection) were included. For the interval between the last cladribine course and vaccination, the date of the last vaccination between the cladribine course and antibody test was used. Antibody titers were determined by a chemiluminescent immunoassay of IgG antibodies against SARS-CoV-2 trimere spike protein (DiaSorin LIAISON). The cut-off for seropositivity was 33.8 BAU/ml and the maximal measurable response 2080 BAU/ml.

The local medical research ethics committee reviewed and approved the study protocol. The need for written informed consent was waived because of the retrospective nature of the study and the use of anonymized clinical data. The primary outcome is the proportion of cladribine-treated patients reaching SARS-CoV-2 seropositivity. The humoral response in patients receiving the vaccination < 18 weeks after the cladribine course were compared against the group who were vaccinated after 18 weeks. Descriptive statistics are used to describe trends in these groups. Data, extracted from local electronic health records, consisted of demographic characteristics, treatment dates, SARS-CoV-2 vaccination/infection dates, antibody response and, if available, lymphocyte counts in serum.

## Results

**Fig. 1:**
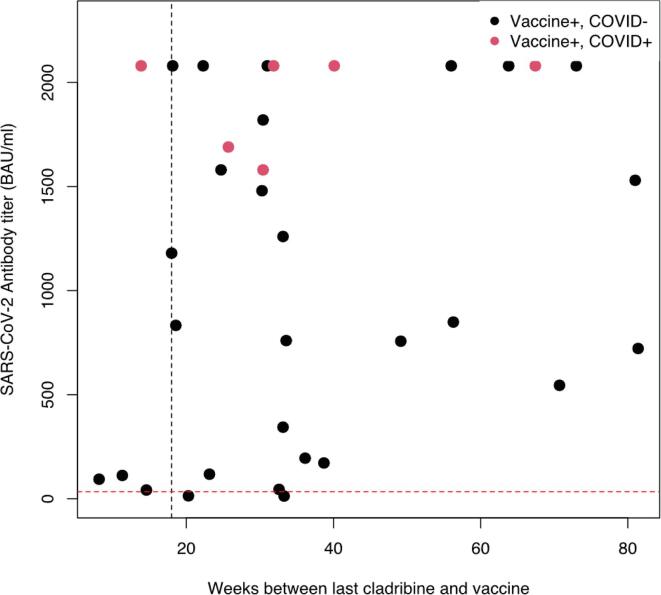
SARS-CoV-2 antibody titer stratified by time between last cladribine and vaccination. Red dashed horizontal line shows cutoff for positive antibody titer (33.8 BAU/ml). Black dashed vertical line shows the recommended minimum of 18 weeks for vaccination after cladribine. (For interpretation of the references to colour in this figure legend, the reader is referred to the web version of this article.)

34 patients met the inclusion criteria. Patient characteristics are shown in Table 1. All patients had relapsing-remitting MS. For 5 patients cladribine was the first DMT they received, 8 patients had had another high-efficacy DMT before cladribine (7 natalizumab, 1 alemtuzumab). The remaining 21 patients had received low- or intermediate efficacy treatment before cladribine. 29 patients were vaccinated after and 5 earlier (mean [range]: 13 [8], [18]) than the recommended 18 weeks (Dutch MS workgroup). In both groups, most patients were vaccinated with a mRNA-vaccine (89 % and 100 %, resp.). Two patients only received one dose (both BNT16b2 mRNA vaccine, both in > 18 weeks group), one of which had a PCR-confirmed COVID infection. Fig. 1 shows the antibody titers of the patients plotted against the number of weeks between the last cladribine treatment course and the last vaccination. Two patients (6 %; 53 and 56 years old) did not reach seropositivity, they were vaccinated 20 weeks (2 doses BNT16b2) and 33 weeks (2 doses ChAdOx1) after their last cladribine course. They had no relevant comorbidities or comedication and no lymphopenia. In the ≤ 18 weeks group, all patients reached seropositivity, with a mean (SD) titer of 702 (9 0 6) BAU/ml. The mean titer was 1207 (7 9 1) BAU/mL in the > 18 weeks group.

**Table 1:** Patient characteristics and vaccination/cladribine/COVID-related baseline data.

**Variable**	**All***^1^*	**>18 weeks**, N = 29*^1^*	**≤18 weeks**, N = 5*^1^*
**Age, years**	52 (41, 56)	52 (42, 55)	39 (29, 60)
**Female**	24 (71 %)	20 (69 %)	4 (80 %)
**Disease duration, years**	11 (4, 17)	11 (6, 17)	9 (3, 19)
**Vaccine**			
BNT16b2	27 (79 %)	23 (79 %)	4 (80 %)
ChAdOx1 nCoV-19	2 (5.9 %)	2 (6.9 %)	0 (0 %)
Heterotropic	1 (2.9 %)	1 (3.4 %)	0 (0 %)
mRNA-1273	4 (12 %)	3 (10 %)	1 (20 %)
**Weeks interval last cladribine vaccination**	32 (23, 47)	33 (30, 56)	14 (11, 15)
**Weeks interval vaccination test**	22 (18, 26)	22 (18, 28)	19 (16, 25)
**COVID-infection**	6 (18 %)	5 (17 %)	1 (20 %)
*^1^* Median (IQR); n (%)

## Discussion

Presently, we investigated the SARS-CoV-2 humoral response post-vaccination in cladribine treated MS patients. The proportion of subjects reaching seropositivity was high (94 %, n = 32) in this study population vaccinated within two years after last cladribine (range 8 – 81 weeks). This is despite the fact that cladribine seems to affect lymphocyte counts until two years after last dose [20]. Current findings are in line with previous studies on the subject by Brill et al (n = 34) [11], Tortorella et al (n = 20) [17], Milo et al (n = 35) [16], Grothe et al (n = 38) [12], Kranjc et al (n = 26) [14] and Achiron et al (n = 48) [4], where all cladribine treated patients reached seropositivity. In the study by Maglione et al. (n = 7) [15], 1 patient did not seroconvert after the first vaccination cycle. In this study, two patients (6 %) did not reach seropositivity. No clear cause could be identified, as patients had no relevant comorbidities or comedication, no lymphopenia and both received two vaccination doses. The seronegative patients were somewhat older (53 and 56), which could be a contributing factor since higher age has been associated with a higher probability of low- or non-response to vaccination [21], [22]. However, the present study finds a similar seropositivity rate as found in studies in large samples from the general population [23].

Limitations of the present study include a small sample size due to cladribine being a second-line and relatively new treatment in MS. Additionally, the interval between vaccination and the antibody-test varied, possibly influencing the titers, but was similar between the ≤ 18 and > 18 weeks group (resp. median of 19 and 22 weeks).

This study confirms findings of earlier reports that MS patients treated with cladribine have an adequate humoral response after vaccination for SARS-CoV-2. In conclusion, our results support earlier studies reporting effective immunization after COVID-19 vaccination in RRMS patients treated with cladribine. Additionally, patients vaccinated before the recommended interval of 18 weeks after last dose of cladribine appear to show successful immunization as well. Further investigation of earlier vaccination may allow a shorter interval between cladribine treatment and COVID-19 vaccination.

## Funding sources

This research did not receive any specific grant from funding agencies in the public, commercial, or non-profit sectors.

## Author contributions

OG, SK, RH conceptualized this study. TK collected the data. DK and MM analyzed the data and drafted the manuscript. All authors critically reviewed, provided feedback and approved the manuscript.

## Declaration of competing interest

The authors declare that they have no known competing financial interests or personal relationships that could have appeared to influence the work reported in this paper.

MM, DK, SK, TK, OG have nothing to disclose; RH received institutional research grants and fees for lectures and advisory boards from Biogen, Merck and Genzyme-Sanofi.

## Data Availability

Data will be made available on request.
